# Osteopetrosis (Marble Bone Disease): A Rare Disease in Children

**DOI:** 10.5005/jp-journals-10005-1115

**Published:** 2011-04-15

**Authors:** Raghunath Reddy MH

**Affiliations:** Former Professor and Head, Department of Pedodontics and Preventive Dentistry, SJM Dental College and Hospital Chitradunga, Karnataka, India

**Keywords:** Osteopetrosis, Marble bone disease, Osteopetrosis tarda, Albers-Schonberg disease.

## Abstract

Osteopetrosis is a group of diseases that affects the growth and remodeling of bone and characterized by over growth and sclerosis of bone, with thickening of the bony cortices, abnormal dental development and narrowing of the marrow cavities throughout the skeleton. It is an uncommon disease of unknown cause. A 5-year-old boy was suffering from infantile (severe form) osteopetrosis with cardiac enlargement, severe anemia, hepatosplenomagaly and radiographs showed generalized increase in bone density (chalky white), narrowing of skull base is reported here.

## INTRODUCTION

Osteopetrosis is a disorder characterized by defective or absent osteoclasts, the cells that resorb bone was first described in 1904 by Heinrich Albers, Schenberg. The name was given by Karshner in 1926.^1,2^ In healthy bone, a steady state is achieved in which the production of bone by cells called osteoblasts is balanced by bony resorption by osteoclasts.

The dysfunctional osteoclasts that are observed in os-teopetrosis result in bony overgrowth, leading to bones that are abnormally dense and brittle.

Osteopetrosis is generally subdivided clinically into benign dominantly inherited form and malignant recessively inherited form. Benign form is the most commonest, usually develops later in life and is less severe. Approximately, half of the patients are asymptomatic and the diagnosis is made incidentally or is based on family history. Many patients have bone pain and are fragile, might fracture easily. Other manifestations include visual impairement and psychomo-tor retardation. Physical findings are related to bony defects and include short stature, frontal bossing, a large head, hepatospenomegaly.

The most severe form of osteopetrosis is termed infantile (malignant) osteopetrosis is an autosomal recessive mode of transmission. This form present at birth or develops in early childhood, adolescence or young adult life. If untreated, infantile osteopetrosis usually results in death by the first decade of life. The disease is severe and debilitating. Patients have symptoms of neurologic and hematological derangements, optis atrophy, severe anemia, bleeding, or infection, blindness, deafness, multiple fractures of the long bones with resulting deformity, hepatosplenomegaly, facial palsy, hy-drocephalus, possible mental retardation and osteomyelitis. Increased bone density of cortical bone and club - shaped appearance of the long bones may be discovered incidentally on X-rays.^3,4^

This form is also called malignant osteopetrosis, not because of a relationship to cancer but because of the severity of the disease.^5,6^

This disorder is inherited in an autosomal recessive pattern, which signifies that both parents are unaffected carriers. When these parents have children, the chance that a particular child will have the disease is one in four.

Infants with osteopetrosis have early loss of vision and also have hearing losses. Because osteoclasts are absent or dysfunctional, the bone marrow cavity in which blood cells are produced does not form normally. A severe form of osteopetrosis with manifestations in the new born and a progressive course leading to death at an early age is called osteopetrosis with precocious manifestations.

### Oral Manifestations

The medullary spaces of the jaws are remarkably reduced in both dominant and recessive type and there is marked predilection for the development of osteomyelitis.

Fracture of the jaw during tooth extraction may also occur without undue force. Teeth are of defective quality, enamel hypoplasia, microscopic dentinal defects and arrested root development, retardation of tooth eruption due to the sclerosis of bone all having been reported.^7^

Treatment strategies include correcting anemia thrombocytopenia and treating infections. Bone marrow transplant and splenectomy may be useful in some patients. Optimal transplants require donor marrow from a sibling. Corti-costeroids, high dose calcitriol have been helpful in the treatment of osteopetrosis. Complications of osteopetrosis can be reversed or prevented in early life by bone marrow transplantation.^8^ Clinical studies have shown that regular administration of interferon-gamma-1b can significantly slow the progression of the disease and reduce infection rates, with few serious side effects.^9^

## CASE REPORT

A 5-year-old male patient visited Department of Pedodontics and Preventive Dentistry, SJM Dental College and Hospital, Chitradurga with a chief complaint of bleeding from the gingiva in relation to lower left back region since week.

On clinical examination, the following observations were made. Dolicocephalic head, exopthalmus, frontal bossing, hypertelerism, depressed nasal bridge, hypoplastic maxilla, mandibular prognathism and the patient was partially mentally disabled. The physical status of the patient was poor.

The oral findings showed presence of 55, 54, 53, 52, 62, 63, 64, 74, 75, 83, 84 teeth, swollen gingiva and mobility in relation to 74, deep palatal vault, spacing between teeth.

Patient medical reports revealed that, boy was alright at first year of age, bleeding from the nose on and off since last 1 year. There was 5 to 10 cc of loss of blood every time. Repeated chest infections, diminision of vision since 3 years, fever, cough, patient was having hurried breathing each time and subsides after treatment and low set ears but no history of loss of hearing. Depressed nasal bridge, mi-crognathia, short fingers, flat fleet, pigeon chest deformity, distended abdomen, enlarged spleen, shifted trachea toward left, elongated skull.

**Fig. 1:**
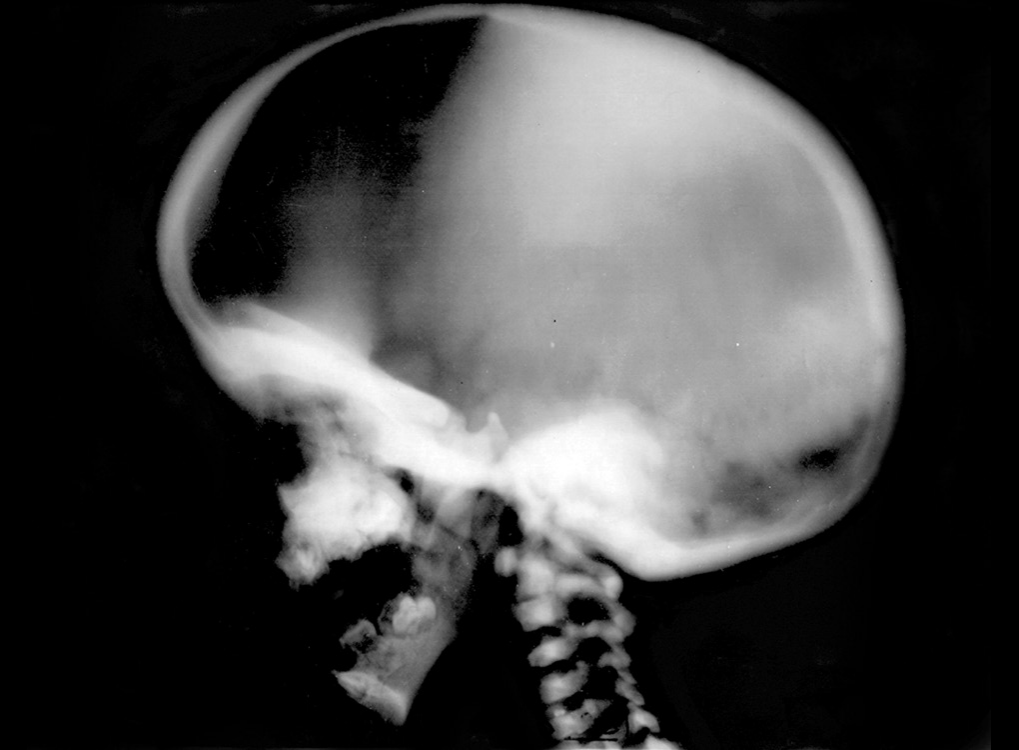
Skull vault showed dense bone with narrowing of skull base and elongated head

**Fig. 2:**
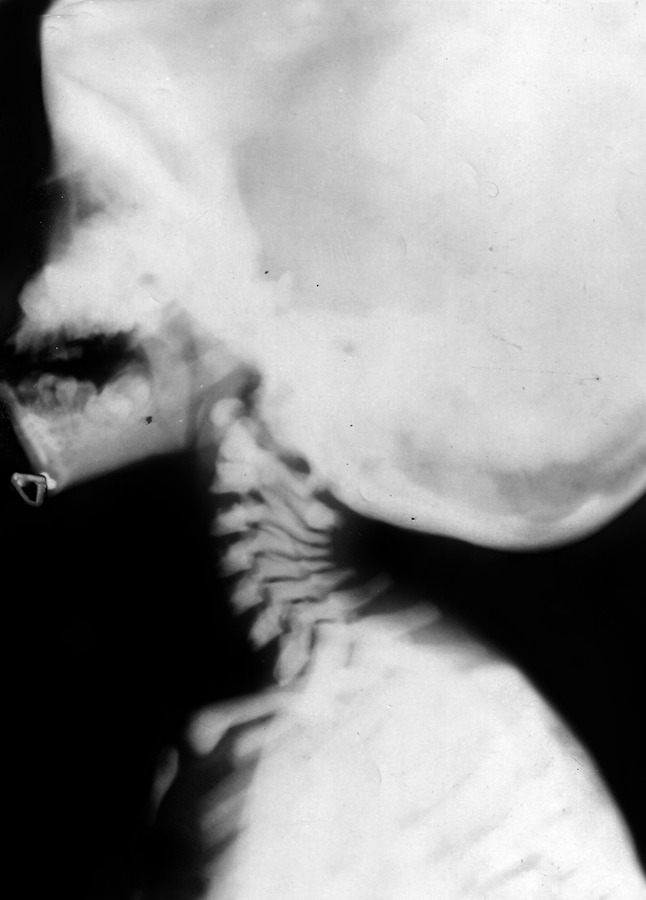
Cervical spine lateral view showed generalized increase in bone density (Chalky white) and skull is looking big

**Fig. 3:**
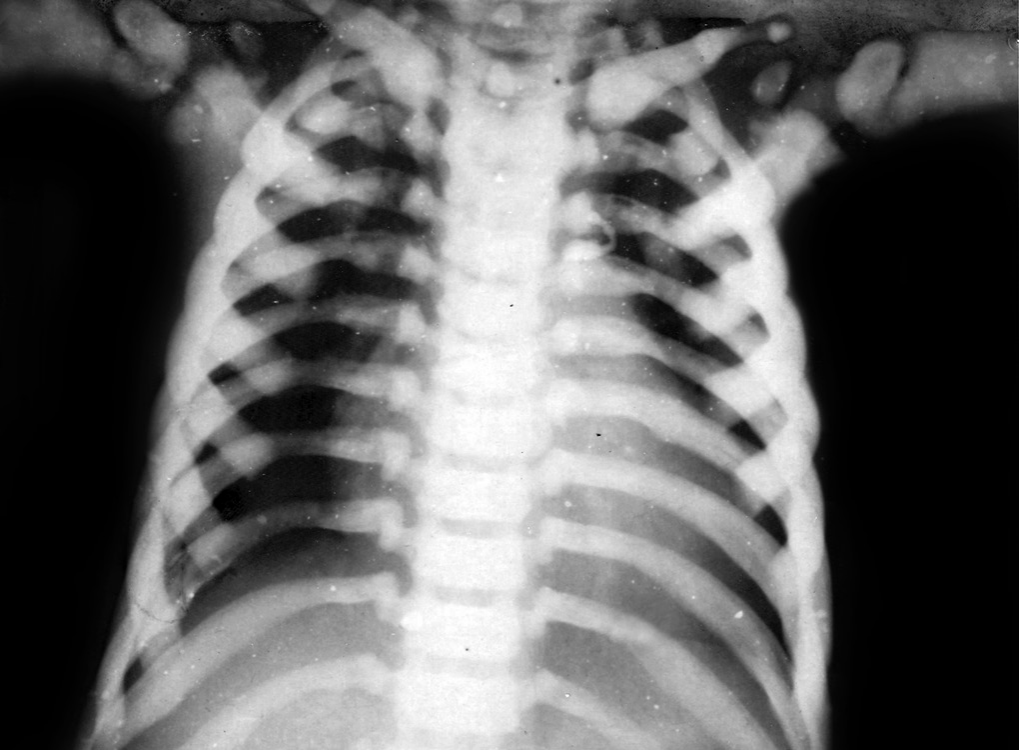
Chest (PA view) showed increased radiopacity of the skeletal bone of the chest and enlargement of cardia

**Fig. 4:**
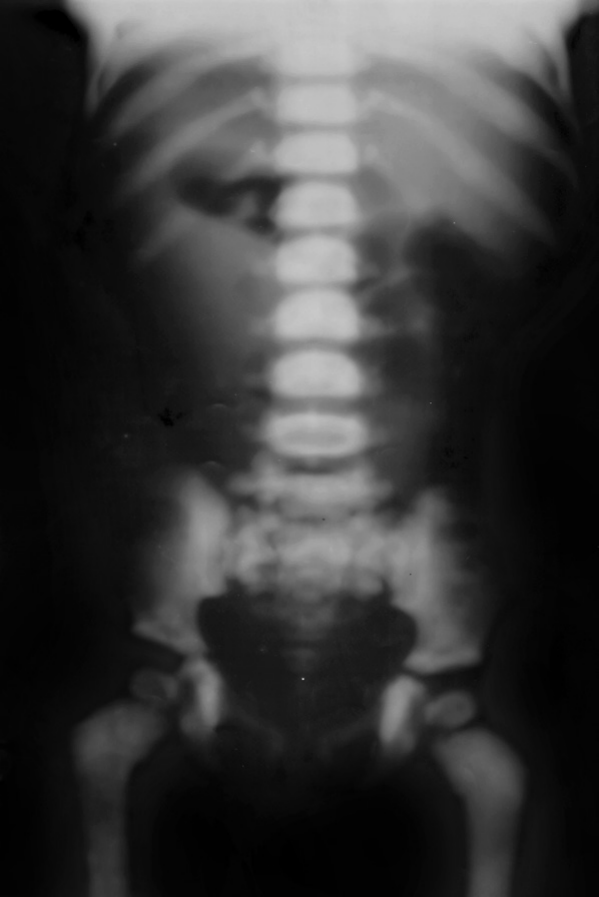
Pelvic lumbo sacral bone: All bones are dense, incomplete fracture of metaphyseal ends of both femoral

Family history was not significant. Active movements of the boy were restricted because of dimension of vision as reported by family members.

Blood investigation reports showed following readings.

Hemoglobin - 4.3 gm, packed cell volume - 20%, reticulocyte count - 4.0%, white blood cells - 7600 cells/ cu mm, polymorphs - 27%, lymphocytes - 52%, monocytes - 01%, eosinophils - 20%, coagulation time - 4 mins, bleeding time - 3 mins.

Patient was diagnosed as suffering from microcytic hypochromic anemia with eosinophilia.

Radiological findings showed generalized increase in bone density (Chalky white) with narrowing of skull base. Enlarged cardia and skull. Increased radiopacity of the skeletal bone of the chest. Cervical vertebra and disk space are normal. Lungs are clear.

## DISCUSSION

The benign form appears later in life and is less severe. The severe form is invariably fatal early in life. The malignant form seen in infancy or develops in early childhood. The os-teopetrosis results from a generalized accumulation of bone mass that is secondary to a defect in bone resorption. This defect prevents the normal development of marrow cavities, the normal tubulation of long bones and the enlargement of osseous foramina.^10^

In the present case report, the symptoms started after first-year of life. Patient had repeated bleeding from the nose, dimension of vision gradually, chest infection off and on, severe anemia, hepatosplenomagaly and elongated head. The radiographs showed generalized increased in bone density (Chalky white), skull and cardia enlargement.

Loria-Cortes et al and Nussey reported cases of osteopetrosis due to consanguineous marriages.^3^ Tips and lynch found frequent affection of siblings on their study while parents were unaffected.^11^ Enell and Pehrson have also analyzed the pedigree chart of three cases of osteopetrosis born of consanguineous marriages.^12^ In our case evaluation, there was no evidence of osteopetrosis in family members examined. The role of consanguinity in osteopetrosis still remains hypothetical. Although parents may be consanguineous, neither malignant osteopetrosis is sporadic and seldom familial, nor are milder variants found in the same family. The severity of the disease status in children in terms of hematological problems, fragility of bones in terms of incidence of fracture and chance of the next issue being similarly affected needs to be evaluated.

## CONCLUSION

Osteopetrosis is a rare congenital disorder. It strikes one in every 200,000 children. Early diagnosis and treatments like controlling infections and bone marrow transplantations should be done in these children to prevent further serious complications. A full mouth rehabilitation program must be carried out for children suffering with this type of disorders.
